# Motivation for postgraduate studies by nonacademic optometrists: A case study at a South African university

**DOI:** 10.4102/hsag.v27i0.1901

**Published:** 2022-09-26

**Authors:** Rekha Hansraj, Nishanee Rampersad

**Affiliations:** 1 Discipline of Optometry, School of Health Sciences, College of Health Sciences, University of KwaZulu-Natal, Durban, South Africa

**Keywords:** optometry, postgraduate, academia, research, supervision, optometrist, institutional reputation, postgraduate studies

## Abstract

**Background:**

An increasing number of optometrists who are nonacademics are pursuing postgraduate studies, despite such qualifications traditionally being more relevant to an academic rather than a clinical setting.

**Aim:**

This study set out to explore possible reasons for the increase in postgraduate studies by nonacademic optometrists and their experiences thereof.

**Setting:**

Postgraduate optometry students who were registered at a selected South African university within the period 2010–2020 participated in the study.

**Methods:**

A descriptive cross-sectional research design was used, which entailed an online survey and a follow-up questionnaire focused on the motivation for postgraduate studies, choice of institution, research area, expectations and outcomes and supervision received. Convenience sampling was used to recruit the participants, and data were analysed with descriptive statistics.

**Results:**

Responses were received from 54 optometrists who were pursuing postgraduate studies but not currently in academia. The main reasons reported for enrolling in postgraduate studies were interest in research (69.2%) and academia (61.5%). Institutional reputation was the reason provided by 48.1% of respondents for their choice of institution for their postgraduate studies. Fifty per cent of respondents opted for research in the public health field. To be a better researcher was the most common expectation (88.5%), with 73.1% indicating achieving this outcome. Most respondents indicated that they had received constructive and timeous supervision during their studies.

**Conclusion:**

Interest in research and academia are important factors influencing nonacademic optometrists to pursue postgraduate studies; however, the postgraduate experience is likely to also facilitate development of other professional and clinical skills.

**Contribution:**

This study provides an insight into the motivation and experiences of non academic optometrists who pursue postgraduate degrees.

## Introduction

Optometry is a healthcare profession that seeks to ensure the visual and ocular well-being of individuals by providing care related to refraction and dispensing, diagnosis and management of ocular disease and the provision of vision therapy and rehabilitation services (*Health Practitioners Competence Assurance Act* No. 56 of 1974). Globally, to qualify as an optometrist, training in many countries, including the United Kingdom (UK), United States of America (USA), Australia, New Zealand and India, involves a minimum of four years at a college or university (Van Staden et al. 2019) to obtain a bachelor’s degree entitled ‘Doctor of Optometry’ and designation of the ‘doctor’ title. However, this basic degree and title is only conferred after a minimum of 6 years of training in countries such as Nigeria and Ghana. In South Africa, the minimum qualification required to practice as an optometrist registered with the relevant professional body is the Bachelor of Optometry (BOptom) degree, which is also over a minimum period of four years but without the ‘doctor’ title.

Factors influencing the choice of a career in optometry include the desire to help others, professional prestige, employment opportunities and a balance between work and family life (Abhilasha & Kulkarni 2020; Boadi-Kusi et al. 2015; Mashige & Oduntan 2011; Osuagwu et al. 2014). These factors are similar to those reported in other health science disciplines (Keshishian et al. 2010) and suggest a leaning towards working with people in a clinical environment. Previous studies (Abhilasha & Kulkarni 2020; Boadi-Kusi et al. 2015; Keshishian et al. 2010; Mashige & Oduntan 2011; Osuagwu et al. 2014) have focused only on the undergraduate student choice process in optometry but not on the postgraduate optometrist. This would be of interest particularly as universities in South Africa and elsewhere place greater emphasis on recruiting postgraduate students (Cobbing et al. 2017; Manyike 2017; McCallin & Nayar 2012).

In South Africa, with the BOptom being a four year 480-credit professional degree, should an optometrist wish to pursue postgraduate studies, the next degrees would be Master of Optometry (MOptom), followed by a Doctor of Philosophy (PhD). This progression through postgraduate qualifications in optometry is similar in other countries, as is evident in the websites of various institutions in the UK, USA and Australia. Postgraduate research degrees in health professions have been perceived at times to be more relevant to and a requirement for an academic rather than a clinical setting (Beeston, Rastall & Hoare 1998; Myint et al. 2014), thus denoting an intention to pursue an academic career (Kearney 2008). This may be somewhat the case for optometry in SA, as a BOptom degree is the minimum qualification required for clinical practice, and it also enables graduates to practice internationally following completion of other requirements, for example, a qualifying competency assessment or examination as guided by the relevant regulatory body. This is in contrast to other disciplines such as speech–language pathology, where a master’s degree is the minimum requirement to practice as a clinician in the USA (Sebothoma, Masuku & Moroe 2021). In medicine and nursing, additional qualifications can enable higher earnings, the likelihood of a promotion and status enhancement (Havenga & Sengane 2018; Sandhu 2018), which does not appear to be the case in other allied health professions, including physiotherapy (Cobbing et al. 2017) and optometry.

More recently, an increase in postgraduate enrolments has been observed in health disciplines for a myriad of reasons (Cobbing et al. 2017; Havenga & Sengane 2018; Hoffman & Julie 2012; Honey, North & Gunn 2006), including the achievement of personal goals together with broadening the knowledge base that translates into improved patient care, as reported by speech language pathologists, audiologists and physiotherapists in South Africa (Cobbing et al. 2017; Sebothoma et al. 2021). In addition, enhancement of clinical decision-making skills, critical thinking and personal careers were also reported by nurses in Australia as motives for pursuing postgraduate education (Ng, Eley & Tuckett 2016). Interestingly, Jasińska-Stroschein, Kurczewska and Orszulak-Michalak (2017) reported that the motivating factor for Polish pharmacists pursuing postgraduate studies was to secure promotions. With the expanding scope of optometry in South Africa, completion of postgraduate courses towards diagnostics and therapeutics certification will pose an advantage to graduates in the clinical setting more so than a masters or PhD degree. Considering that most of optometrists are practising either in the public or private sector and only 1% are in academia, the need to explore the motivation for postgraduate qualification is evident. No research has to date explored the motivation or experience of nonacademic optometrists choosing to undertake postgraduate degrees. Thus, information on the motivation and experiences of the South African postgraduate optometrist would be particularly useful for tertiary institutions in SA in guiding future recruitment and marketing strategies, as well as help in the planning of postgraduate degree offerings.

## Methodology

### Research design

A descriptive, cross-sectional research design was utilised.

### Instrumentation

An online questionnaire (Questionnaire 1) was developed specifically for this study by the authors using Google Forms (with 32 closed-ended forced choice questions and a single open-ended question for additional comments) and a follow-up questionnaire (Questionnaire 2) with open-ended questions only. A questionnaire, administered online, was decided upon as the data collection method, as it appears to be the preferred method in educational research, particularly for their quicker responses, lower administrative costs and higher response rates than paper-based surveys (Saleh & Bista 2017).

The questionnaire was divided into three sections. Section A ascertained the biographical data of the respondents include age, gender, race, nationality, employment sector, alma mater, qualifications and interest in academia. Section B determined details of the current and previous postgraduate studies of the respondent, including institution at which they registered, choice of institution, focus area of postgraduate studies, motivation to register for postgraduate studies and outcomes of their postgraduate studies. Section C focused on the experiences, including challenges, of the respondents during their studies based on their responses on a five-point Likert scale, ranging from strongly agree to strongly disagree, to statements structured around administrative issues, affordability and supervision. Questionnaire 2 was used to better understand the reasons and explanations for the trends being observed and noted in the online questionnaire (Cohen, Manion & Morrison 2007). Questionnaire 1 was piloted on five optometrists who were not necessarily registered for postgraduate studies but were not part of the main study, to establish face and content validity.

### Sampling

For Questionnaire 1, convenient sampling was used to recruit all optometrists (*n* = 95), of any age, gender, race or marital status, registered at a selected South African university for postgraduate studies during the period 2010–2020.

Academic staff in the discipline of optometry at the time of the study were contacted and asked to share the purpose of the study with their postgraduate students for the period 2010–2020 and obtain consent to share their details to be contacted for participation. Using these details, subjects were invited personally via e-mail to participate in the study. The links to provide informed consent and to the online survey were included in the e-mail. The degrees that the respondents had already completed or were currently registered for included a Masters in Optometry, Masters in Public Health (PH), Masters in Business Administration, PhD (Optometry), PhD (PH) and a postgraduate diploma in health economics. Optometrists who held full-time academic positions at the time of sending out the online questionnaire were excluded from the study. In the online questionnaire, respondents were asked if they would like to participate in a follow-up questionnaire to better explain the reasons for their responses in the online questionnaire. While five respondents from the initial cohort indicated their willingness to participate in the follow-up questionnaire, only two were still available and willing to complete the follow-up questionnaire.

Data were captured and analysed using the Statistical Package for Social Sciences (SPSS) version 27 using descriptive statistics and thematic content analysis. The chi-square test was used to determine associations at a 95% confidence level.

### Ethical considerations

The tenets of the Declaration of Helsinki were adhered to during this study and included obtaining informed consent from subjects prior to their participation. Ethical clearance to conduct this study was obtained from the Humanities and Social Sciences Research Ethics Committee at the University of KwaZulu-Natal (reference number: HSS/1410/017).

## Results

### Characteristics of the sample

**FIGURE 1:**
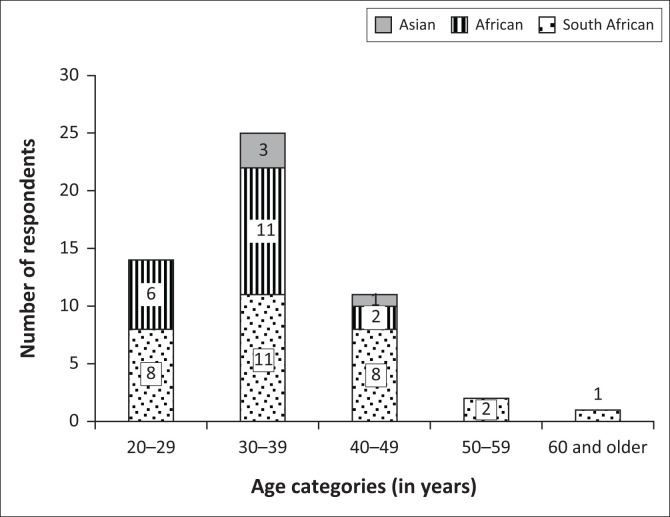
Age and nationality categories of respondents.

Of the 95 postgraduate optometry students to whom the online survey was e-mailed, 54 responded, giving a response rate of 57%. However, only 53 responses were included in the analysis, as one respondent had recently become a full-time academic. Of these respondents, more were female (56.6%) than male (43.4%). At the time of data collection, 55% were registered for a MOptom degree, 30.6% for a PhD degree and the remainder (14.4%) having completed either a MOptom (10.3%) or PhD (4.1%) degree. One respondent had also completed a Master’s in Business Administration (MBA) and another a postgraduate diploma in health economics. Based on nationality, just over half of the respondents (*n* = 30; 56.6%) were South African, with 35.8% (*n* = 19) being from other African countries including Cameroon, Ghana, Malawi, Mozambique, Nigeria, Sudan, Swaziland and Tanzania and a small percentage (7.6%) from Asian countries, including India (*n* = 1), Nepal (*n* = 2) and Malaysia (*n* = 1). The age and nationality of the respondents are further illustrated in Figure 1.

Table 1 describes the respondents according to gender, marital status and employment sector. The majority of respondents were employed in the private sector (62.2%) and most commonly served as the sole proprietor of a nonfranchise practice (28.6%). A marginally larger number of respondents in the entire sample were either single or divorced (*n* = 29) compared with those who were married (*n* = 24). The 7.6% of respondents who had indicated research or university as their employment sector were optometrists who served as clinical supervisors at the university-linked clinics.

**TABLE 1:** Cross-tabulation of gender, marital status and employment sector of respondents (*n* = 53).

Gender of respondent	Employment sector												Total	
	Private		Public		Research		Full-time student		University		Both private and public			
	*n*	%	*n*	%	*n*	%	*n*	%	*n*	%	*n*	%	*n*	%
**Female**														
Marital status														
Married	5	-	3	-	1	-	0	-	1	-	1	-	11	-
Single	12	-	4	-	0	-	1	-	0	-	1	-	18	-
Divorced	1	-	0	-	0	-	0	-	0	-	0	-	1	-
Total	18	-	7	-	1	-	1	-	1	-	2	-	30	-
**Male**														
Marital status														
Married	9	-	2	-	1	-	-	-	-	-	1	-	13	-
Single	5	-	3	-	1	-	-	-	-	-	0	-	9	-
Divorced	1	-	0	-	0	-	-	-	-	-	0	-	1	-
Total	15	-	5	-	2	-	-	-	-	-	1	-	23	-
**Total**														
Marital status														
Married	14	-	5	-	2	-	0	-	1	-	2	-	24	45.3
Single	17	-	7	-	1	-	1	-	0	-	1	-	27	50.9
Divorced	2	-	0	-	0	-	0	-	0	-	0	-	2	3.8
Total	33	62.2	12	22.6	3	5.7	1	1.9	1	1.9	3	5.7	53	100

Most of the respondents had obtained their BOptom degree from the selected university (40.4%), while three (5.8%) had obtained their bachelor’s degrees in Ghana and four (7.7%) in Nigeria.

The majority of respondents (71%) indicated an interest in obtaining a position in academia. Neither gender (χ^2^ = 1.826; *p* = 0.401) nor employment sector (χ^2^ = 2.881; *p* = 0.984) was associated with interest in academia, although age showed a marginally significant association (χ^2^ = 15.275; *p* = 0.054), with the younger optometrists more likely to display this interest.

A similar percentage (71.2%) indicated that they had colleagues not in academia who had obtained postgraduate degrees; however, no association was observed between having colleagues in academia and a respondent’s own interest in academia (χ^2^ = 5.530; *p* = 0.63).

Both respondents who participated in the follow-up questionnaire were male, under 30 years of age and employed in the public sector. Respondent 1 (R1) was Ghanaian, and Respondent 2 (R2) was South African. Both indicated an interest in academia.

### Reasons for undertaking postgraduate studies

**FIGURE 2:**
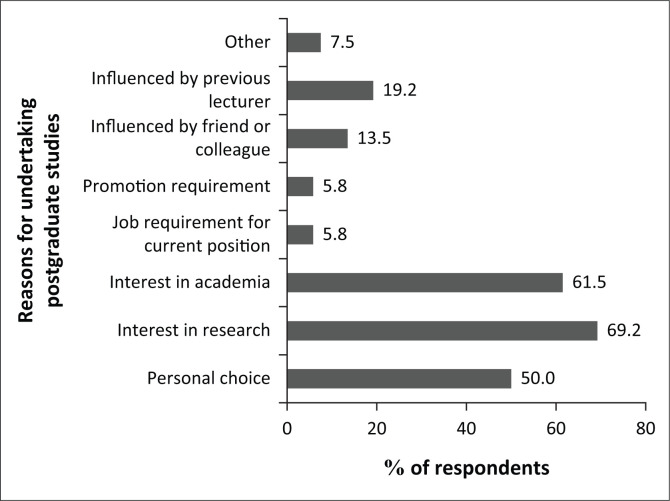
Reasons for undertaking postgraduate studies.

Reasons for undertaking postgraduate studies, as reported by the respondents, included having an interest in research, as reported by 69.2% of respondents, followed by interest in academia (61.5%) and then personal choice (50%) (Figure 2). Respondents were allowed to choose multiple options for this question.

Reasons for the higher percentages interested in research or in an academic setting relates to perceived higher intellectual stimuli when compared with the clinical environment. That further study while in the clinical setting, poses no advantage to career advancement in a clinical setting, was highlighted by R1:

‘I felt that the clinic schedule was boring, with routine procedures and not challenging. I also had a soft spot for research. Although one can be at the clinic and still do research, it is not rewarding because it will not be used in promotions, among others in my home country. Hence, I opt to go to academia.’ (R1, male, Ghanaian)

‘I want to be in academia or research. I find it exciting and challenging.’ (R1, male, Ghanaian)

R2 expressed a more philosophical reason:

‘In my community, there have been very few people who have gone to the university, let alone have postgraduate. By pursuing postgraduate studies, I hope that I will inspire the member of my community that is possible.’ (R2, male, South African)

In addition, R1 shared:

‘The more you learn the better you become and the contribute to the health and optometric profession.’ (R1, male, Ghanaian)

While only 19.2% of respondents during the first phase of the study indicated that a previous lecturer had influenced their decision to take up postgraduate studies, it was a major factor for R2, as he stated that:

‘[*H*]aving done research in my undergrad studies really inspired me to pursue postgraduate. I was really inspired by the commitment and the passion of my supervisor.’ (R2, male, South African)

A challenge observed in relation to employment sector was expressed by R2, who is currently the resident optometrist at a public sector community health centre: ‘Having to study while working has been one of the biggest challenges’.

### Choice of institution

The most popular reason for choosing the institution for postgraduate studies was institutional reputation (48.1%), followed by having completed their BOptom degree in the same institution (32.7%) and the preferred supervisor being located at the institution (32.7%) (Table 2).

**TABLE 2:** Reasons for choosing institution for postgraduate studies (subjects could choose more than one option).

Reason for choice of institution	% of respondents
Bachelor’s degree completed there	32.7
A previous qualification completed there	9.6
Only institution offering chosen degree	1.9
Preferred supervisor located there	32.7
Institutional reputation	48.1
Proximity to place of employment	13.5
Recommended by colleague or employer	25.0
No particular reason	5.8
Too expensive to study abroad	5.8
Work there	1.9
Flexibility of programme	1.9
Fees covered by institution for postgraduate studies	1.9
Ease of application and admission process	1.9

Institutional reputation was the influencing factor for both respondents. For example, R1 indicated:

‘Among the few countries running postgraduate degrees in Optometry in Africa, [*name of university*] was at the top in research, publications and renowned personnel. These data were an essential factor in my decision-making.’ (R1, male, Ghanaian)

R2 also expressed similar reasons for choice of institution and indicated:

‘The amount of support, experience, learning that I got from my undergrad research supervisor inspired me to pursue postgraduate studies at [*name of university*].’ (R2, male, South African)

‘The knowledge I got from the undergraduate made me want to be keep to explore optometry outside the normal parameters on what most optometrist do when they graduate.’ (R2, male, South African)

### Focus area of research study

**FIGURE 3:**
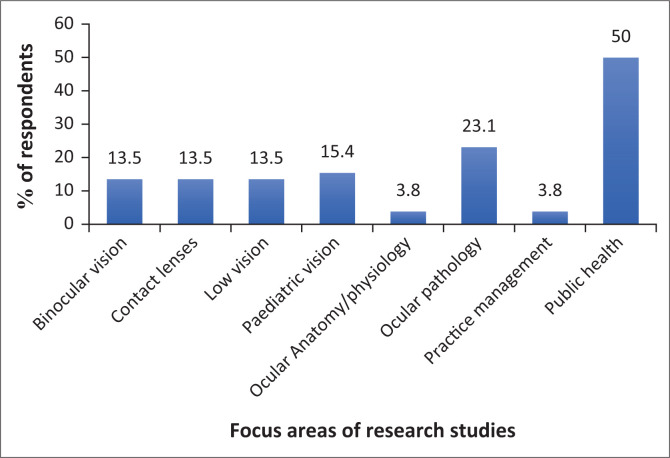
Focus areas of research studies being undertaken for the postgraduate qualification.

Most of the research studies being undertaken were focused on public health (50%). Fewer studies were being undertaken in the other focus areas outlined in Figure 3. Some research studies straddled more than one focus area.

The focus area chosen for postgraduate studies can further professional development as a clinician as expressed by R2:

‘There is a vast difference between what you learn during your undergraduate and when you are doing the postgraduate and more when you focus on that field that you are more interested to.’ (R2, male, South African)

‘I would encourage every optometrist to do research, as it expands how one think(s) in terms of analysing the clinical results, conducting case history and clinical assessments.’ (R2, male, South African)

### Expectations and outcomes of postgraduate studies

Table 3 outlines respondents’ expectations of postgraduate studies in optometry together with the outcomes some experienced after obtaining their postgraduate qualification. The most common expectation was to be a better researcher (88.5%), with a similar outcome of becoming a better researcher expressed by the majority of respondents (73.1%). The Fischer’s exact test showed a significant correlation between expectations and outcomes as indicated by the *p*-values.

**TABLE 3:** Expectations and outcomes of undertaking postgraduate studies by optometrists.

Expectations	%	Outcomes	%	Fischer’s exact test (*p*-value)
Make me a better practitioner	63.5	Made me a better practitioner	53.8	0.009
Make me a better researcher	88.5	Made me a better researcher	73.1	0.010
Improve my chances of getting into academia	55.8	Improved my chances of getting into academia	46.2	0.000
Improve my status among my patients	15.4	Improved my status among my patients	26.9	0.002
Improve my status among my friends and colleagues	11.5	Improved my status among my friends and colleagues	19.2	0.004

However, the expected benefit and actual outcome was not only in research, as observed in Table 3, as both respondents also highlighted the benefits in various skills. In this regard R1 indicated:

‘I have learnt a lot. Pursuing a postgraduate study has improved my writing, problem-solving and management skills’ …. ‘Professionally, I have had contacts with scholars I never dreamt of meeting or interacting within this life.’ (R1, male, Ghanaian)

In addition to these skills, R2 stated:

‘I have also observed that the more you learn, the better you become as a person and clinician.’

The majority of respondents strongly agreed (48.1%) or agreed (34.6%) with the statement that they would encourage other optometrists to further their postgraduate studies even if they are not going into academia. This was also indicated by the two respondents, where R1 expressed:

‘Others who would like to advance their knowledge in the discipline too would be encouraged, because the study is not only about research but capacity-building, knowledge of new developments, especially in coursework and coming up with innovations.’ (R1, male, Ghanaian)

In the same way, R2 expressed:

‘I would encourage everyone to pursue postgraduate studies. Postgraduation allows you to think critically and also contributes to your profession.’ (R2, male, South African)

The following comment was observed by another respondent in the ‘other comment’ section of the online questionnaire:

‘Anecdotally, I have found that many of my peers believe that, especially in private practice, a postgraduate qualification in optometry is not relevant and will not help further one’s career. If they do consider furthering their studies, they are more inclined towards an MBA or other business-related degree.’

### Administrative issues

**FIGURE 4:**
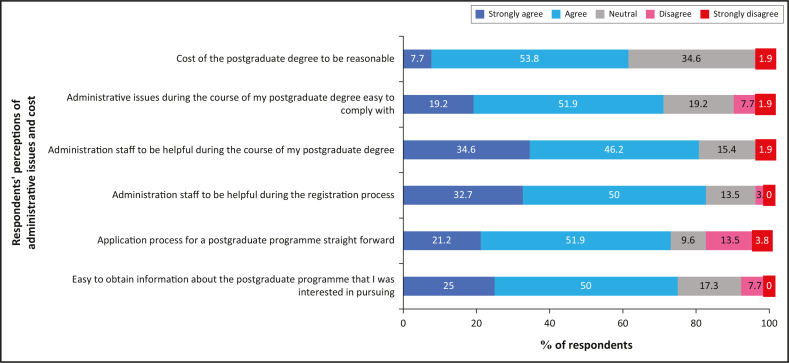
Respondents’ perceptions of administrative issues and cost during their postgraduate studies.

Figure 4 outlines the respondents’ perceptions of the administrative issues linked to their postgraduate degree. Most (61.5%) perceived the cost to be reasonable, with only two respondents disagreeing.

While Figure 4 suggests minimal challenges with administrative issues during postgraduate studies, challenges may have arisen with other processes including application for ethical approval and acquisition of scholarship funds as expressed by R1:

‘A major challenge is an ethical clearance. Ethical issues are important, but sometimes the process takes longer. For instance, students outside South Africa have to seek ethical approval from gatekeepers in home countries to conduct their studies before applying for BREC clearance. But it takes months for Biomedical Research and Ethics Committee [*BREC*] to provide an expedited clearance. Also, funding is another major challenge. During my masters, I received a CHS [*College of Health Sciences*] scholarship, but I could notaccess the funds for logistic purposes.’ (R1, male, Ghanaian)

Other challenges observed from respondents to the online questionnaire included ‘statistical support lacking’ and ‘challenging to balance work and academic life’.

### Supervision

Most respondents (59.6%) strongly agreed or agreed (34.6%) that the feedback received from their supervisors was constructive. Similarly, the majority (51.9%) strongly agreed and 32.7% agreed that the feedback was received timeously. This was further noted by the comments expressed by both respondents regarding overall positive experience with supervision. R1 indicated:

‘The supervision was outstanding. My supervisor was prompt in providing constructive feedback.’ (R1, male, Ghanaian)

R2 had similar experiences and commented that:

‘Having worked with my master’s supervisor during undergraduate studies made it easy for me to understand what was wanted, however, with a little more intensity. The experience was constructive, and it has enabled me to be a better clinician as it has expanded the way I manage and work with patients. The feedback was timeous.’ (R2, male, South African)

Regarding challenges with supervision, R1 expressed:

‘A major issue was when the supervisor disagreed with a co-supervisor, which would delay the response time and affect(ed) the work schedule.’ (R1, male, Ghanaian)

The following comment was also observed from one of the other respondents in the section ‘Other comment’: ‘Statistical support was sadly lacking’.

### Additional recommendations to improve experience of postgraduate students in optometry

The following recommendation was provided by R1:

‘I want the school to create a platform where distant students can interact and share and solve issues about their studies. At the moment, I do not know any postgraduate students at the school.’ (R1, male, Ghanaian)

While R2 again was more philosophical in the response as advice to prospective postgraduate student on how to stay motivated:

‘Choose something that you are passionate about that even when you’re bored you’d do it. Just because it’s not easy doesn’t mean it’s not possible.’ (R2, male, South African)

## Discussion

Recently, an increasing number of nonacademic optometrists are opting to pursue postgraduate research degrees. However, the motivations behind enrolling for and the experiences during their postgraduate studies have remained largely unexplored in the discipline of optometry, and yet this information could enhance the recruitment for and delivery of postgraduate degrees by the discipline and university. An interest in research, particularly in the public health field, development as a researcher, as well as in pursuing a career in academia appear to be the motivations behind taking up postgraduate studies by nonacademic optometrists. The enhancement of professional and clinical skills has also been observeds to be an outcome of postgraduate studies. An overall positive experience with supervision and administration during the postgraduate degree was reported.

A satisfactory response rate of 57% was achieved for the online questionnaire and included a marginally higher number of female respondents (56.6% vs. 43.3% male respondents) in contrast to the findings of Saleh and Bista (2017), who reported that men are more likely to respond to online surveys, particularly if they received a reminder to participate. However, Saleh and Bista (2017) did report that a gender difference in response may cease to exist when there may be the possibility of a vested interest in the study. The gender difference of respondents in this study may have also been influenced by a higher number of women in the health science field (Abhilasha & Kulkarni 2020; Kelly 1989) and the gender demographics of the targeted sample.

The majority of respondents fell into the age category of 30–39 years, in keeping with the expected age for masters and doctoral students being 32 and 36 years, respectively (Mouton et al. 2015), followed by the 20- to 29-year-olds. The age range observed suggests that younger optometrists are more likely to embark on postgraduate studies, possibly because of greater interests in this avenue or because they are still deciding on their career paths, while older optometrists may be more settled in their professional clinical careers. This was similar to the findings of Hoffman and Julie (2012) and Sebothoma et al. (2021) that graduates of between 4 and 5 years were more likely to pursue postgraduate studies. Moreover, while it could have been expected that there would be more respondents that were single or divorced, as these individuals may have relatively fewer responsibilities and more time available for personal development (Sobal & Hanson 2010), only a marginal difference in marital status was observed among the respondents.

The private sector optometrist, particularly one who was a sole proprietor of a nonfranchise practice, was more likely to embark on postgraduate studies than optometrists practising in the public sector. This may be related to time constraints experienced particularly in the public sector settings as a result of comparatively higher patient numbers, as was expressed by one of the respondents. Heavy workloads have been reported as a barrier to embarking on and completing postgraduate studies (Cobbing et al. 2017; Havenga & Sengane 2018; Ng et al. 2016; Sebothoma et al. 2021). On the other hand, the private sector optometrists may be more inclined to having time available for other personal development activities. The lower number of public sector employed optometrists doing postgraduate studies may also be possibly attributed to public sector optometrists seeking clinical development programmes rather that research programmes. These aspects were not investigated in this study and will require further study.

Based on where the respondents had obtained their bachelor’s degree, it was observed that the majority of students would probably return to their ‘alma mater’ to pursue postgraduate studies, as 40.4% of the respondents had returned to their undergraduate institution. In this study, return to the ‘alma mater’ was influenced by previous experience with lecturers and the quality of teaching at the institution, as has also been observed by Jung and Lee (2019). Sebothoma et al. (2021) found that ‘bad experiences’ during undergraduate years made speech–language pathologists and audiologists unwilling to return for postgraduate studies but recommended that further studies should explore what these ‘bad experiences’ actually were. Interestingly, this selected institution also attracted postgraduate students from outside South Africa, particularly Nigeria and Ghana, which could be attributed to a previous professor in the discipline of optometry being from Nigeria. In addition, institutional reputation in research was the most common reason provided for choice of postgraduate institution, with one respondent citing this as an essential factor in this decision as also reported by Mouton et al. (2015). Furthermore, the attraction of students from both the African and Asian continent appears to be because of collaborative efforts between the Department of Optometry at the university and other research organisations. Exploring these factors can guide institutions in developing effective strategies for the recruitment and retention of international postgraduate students.

While interest in research was the most common reason for undertaking postgraduate studies, obtaining a position in academia also appears to be an important motivating factor, particularly for younger (under 40 years) optometrists. Again, this trend could be related to optometrists in this age group still establishing their career paths. Historically, uptake of postgraduate education has been reflective of an intention to pursue a career in academia (Kearney 2008). In this study, just over 50% of the respondents had a similar expectation of postgraduate studies, with just under 50% indicating that the postgraduate qualification had actually improved their chances of obtaining an academic position. Sebothoma et al. (2021) also reported that while 43% of speech–language pathologists and audiologists were pursuing postgraduate studies, only 36% were interested in an academic career. These findings suggest a broader view of the benefits of postgraduate studies by health professionals and that it may no longer be limited to just a means of acquiring a post in academia.

Another important factor appears to be colleagues not in academia who possess postgraduate degrees. This may suggest that a postgraduate degree is seen as a status symbol, as expressed by Dzenga (2021) in a newspaper article. A literature search did not reveal any published study reporting this perception for postgraduate studies; however, the publication by Van Den Bor and Shute (1991) reported that higher education in general is perceived as a status symbol. This was also observed in this study, with some respondents expecting and experiencing an improvement in their status from their patients and colleagues. As no other study has delved into this issue, these findings are hypothetical and should be explored in similar future studies. There was also a perception that research and academia were more stimulating than the clinical environment. In addition, R2 expressed that pursuing postgraduate studies was a means of inspiring younger generations to aspire to greater heights, despite challenging circumstances. This is similar to the ‘fulfilment of a personal goal’ reported as a primary facilitator of physiotherapists taking up postgraduate studies (Cobbing et al. 2017).

Becoming a better researcher was both the expectation of postgraduate studies and the outcome expressed by the majority of the respondents. This finding was different from the main facilitators for pursuit of postgraduate education expressed in other health disciplines (Cobbing et al. 2017; Hoffman & Julie 2012; Jasinkska-Stroschein et al. 2017; Ng et al. 2016; Sebothoma et al. 2021), where the fulfilment of personal goals and broadening of the knowledge base were the main reasons found. Unlike other health disciplines (such as medicine, nursing and pharmacy) that offer coursework and research masters programmes, the current postgraduate optometry programmes in South Africa are full research degrees only. This may explain why respondents in the present study anticipated the main outcome as becoming better researchers. In contrast, Mutula (2011:186) asserted that ‘the perception about the role of postgraduate study is not necessarily research but motivation for better career prospects’. However, while a postgraduate research qualification in optometry does not necessarily spur on the career trajectory in a clinical setting, the development and enhancement of professional development skills, including problem-solving, critical thinking and time management, during the postgraduate studies may improve performance in the clinical environment (Ng et al. 2016). Furthermore, formalised research in any health profession can be expected to broaden the clinical knowledge base of both the researcher and the profession (Adams 2007; Sandhu 2018). This is important as many respondents in this study also had expectations of further development as clinicians following postgraduate studies, although this outcome was observed by comparatively few respondents. This outcome was also expected by other health professionals engaging in postgraduate studies (Cobbing et al. 2017; Hoffman & Julie 2012; Ng et al. 2016; Sebothoma et al. 2021). The expectation of postgraduate studies also allowing for improvement in clinical skills should be factored into the planning of postgraduate courses in future. An additional benefit reported by a respondent was expansion and networking with other colleagues in the field, with subsequent benefit to the profession and public.

Most of the respondents were undertaking postgraduate studies focused on public health. Fewer studies were focused in the other subspeciality areas of binocular vision, paediatric vision, etc. Sebothoma et al. (2021) found that speech language pathologists and audiologists pursued postgraduate studies to develop expertise in specialised areas. In line with the potential for enhancement of clinical skills and professional development, the paucity of postgraduate optometry studies in these subspeciality areas reflected in Figure 3 should be taken into consideration when initiating future studies, which corroborates the assertions of previous studies (Cobbing et al. 2017; Sebothoma et al. 2021). This is of particular importance as healthcare practice in any discipline needs to be evidence-based (Adams 2007; Cobbing et al. 2017; Sandhu 2018), and appropriate research in these fields can broaden the current knowledge base and test newer, more innovative, efficient and cost-effective management strategies.

Respondents expressed an overall positive experience with administrative issues prior to registration and during the course of their postgraduate degrees, in contrast to the reporting of negative experiences of students with postgraduate processes and policies at another South African university (Cekiso et al. 2019). Only two respondents reported the cost to be unreasonable, in contrast to the report by Mutula (2011) that unaffordability was one of the challenges to postgraduate studies in different institutions, and across, varying faculties, in Africa. Operational costs and running expenses were reported as challenges by postgraduate nursing students (Havenga & Sengane 2018; Ng et al. 2016). Postgraduate students in optometry may often be already employed, and therefore the cost of their studies may be more affordable, unlike during their undergraduate training where they may have had to rely on external funding. In optometry, the cost of a postgraduate degree is lower than an undergraduate degree, as the former are pure research degrees with no formal instruction. The fees for a coursework masters may be more expensive than that for a research masters, and this should be considered by postgraduate curriculum planners for future postgraduate optometry degree offerings.

The lack of funding for postgraduate studies was observed as a challenge in the African continent (Mutula 2011) and in other health disciplines in South Africa (Havenga & Sengane 2018). In terms of funding, the CHS at the selected institution offers a postgraduate scholarship that covers the student tuition and operational costs. This initiative is an important incentive for the recruitment of and support mechanism for postgraduate students, noting that the cost was seen as unreasonable by two respondents, with a third of the respondents remaining neutral on this issue. However, administrators must also be aware that access to awarded funds was reported as a challenge. Other processes such as ethical clearance may be posing challenges for candidates, particularly because of the duration for the process to be completed and that non-South African students need ethical approval from relevant statutory bodies in their home countries in addition to the approval from the registering institution’s ethics committees.

The respondents’ experiences with supervision during their postgraduate studies was observed as positive in terms of both constructive and timeous feedback, with the respondents describing the experience as ‘outstanding’, ‘constructive’ and ‘timeous’. This is encouraging as effective, constructive and timeous postgraduate supervision has been observed as a challenge at universities worldwide (Cekiso et al. 2019; Havenga & Sengane 2018; Manyike 2017), with negative supervisory experiences often leading to disillusionment of the postgraduate student. Disagreement between the principal and co-supervisor delaying the progress of work was reported by one of the interviewees as a challenge in respect of supervision in this study and must be taken into consideration for situations where postgraduate students have more than one supervisor.

One of the respondents suggested that a postgraduate student forum may be useful for enhancing and supporting the postgraduate study experience particularly for international students. The CHS at the selected institution has initiated a Doctoral Academy, which is a virtual centre aimed at development and support for PhD candidates. Such collaborative groups have been recommended to improve the learning environment and postgraduate throughput rates at higher education institutions (Cekiso et al. 2019; Havenga & Sengane 2018). In addition, such initiatives may also increase the pool of black academics within health science disciplines, specifically targeting the transformation and decolonisation agendas of higher education institutions in South Africa (Sebothoma et al. 2021).

## Limitations and recommendations

The findings of this study cannot be generalised to all South African optometrists or optometrists elsewhere, as it was limited to only one institution and a selected group of postgraduate optometry students. Furthermore, the study population did not include optometrists who had not registered for postgraduate studies, who may have provided insight into reasons for not considering furthering their studies. Moreover, only two respondents completed the follow-up questionnaire, which could have provided more detailed information to the aspects studied. The poorer response (3.7%, *n* = 2/54) obtained for the follow-up questionnaire that could be explained by time constraints experienced by optometrists working and studying concurrently, as expressed by one respondent and furthermore because of other personal issues, particularly as data collection was undertaken during the COVID-19 pandemic. Vance (2011 cited in Saleh & Bista 2017) termed this as ‘seasonality’, where lower survey responses may be expected when the targeted sample is involved in their own studies. Despite only two responses on the follow-up questionnaire, the perspectives of both MOptom and PhD degrees were provided, as one of them was currently registered for PhD while the other had recently completed the MOptom degree. Future studies should, however, enrol a larger sample of optometry graduates at all South African institutions and use qualitative exploratory research designs and instruments to better understand the experience and process of optometry postgraduate degrees. In addition, studies to explore these issues across all health professions are also recommended (Cobbing et al. 2017).

Postgraduate qualifications by health professionals, including via research degrees, should be considered for both promotion and increased remuneration in both the private and public sector. Employers should consider the benefit of study leave to enable employees to take up postgraduate studies. Coursework masters should be considered in future offerings of postgraduate optometry programmes.

## Conclusion

The significance of this study is that it appears to be the first study that has explored the motivation for the increased uptake of postgraduate research degrees by nonacademic optometrists both nationally and internationally. This avenue of embarking on postgraduate studies in optometry, even by those not pursuing academia, has great potential for expanding the knowledge and evidence base of the optometry profession and professional development, which will have direct beneficial implications for eye care.
